# Supplementary material for: Exploring awareness of hearing loss and ear health in Jordanian adults
Source: PLoS One. 2024 Dec 31;19(12):e0316622. doi: 10.1371/journal.pone.0316622 (PMC11687744; doi:10.1371/journal.pone.0316622)
Supplement: Attachment — Submitted filename: Resposne to Reviewers.doc [file pone.0316622.s002.doc]

November 12, 2024

**Dr. Ateya Megahed Ibrahim El-eglany
Academic Editor, PLOS ONE**

**Dear Dr. Ateya Megahed Ibrahim El-eglany,**

**Manuscript: PONE-D-24-05749**

We would like to thank you for considering our manuscript and we extend our thanks to the Reviewers for their time and very helpful comments. A point-by-point response to the editor’s request and the reviewers’ suggestions is provided below and these changes have been implemented in the revised version of the manuscript. We trust that our revisions are satisfactory.

Thank you again for considering this manuscript for publication in *PLOS ONE.*

Best Regards,

**Corresponding author**

**Dr. Yazan Gammoh**

**Associate Professor, Department of Optometry**

**A-Ahliyya Amman University, Amman, Jordan**

1. **Journal Requirements:**

| **Comment field** | **Original comments** | **Reply by the author(s)** | **Changes done on page number and line number** |
| --- | --- | --- | --- |
| Journal Requirements | When submitting your revision, we need you to address these additional requirements.  1. Please ensure that your manuscript meets PLOS ONE's style requirements, including those for file naming. The PLOS ONE style templates can be found at https://journals.plos.org/plosone/s/file?id=wjVg/PLOSOne_formatting_sample_main_body.pdf and https://journals.plos.org/plosone/s/file?id=ba62/PLOSOne_formatting_sample_title_authors_affiliations.pdf | Thank you.  The affiliation of the authors was updated to match the journal’s formatting requirements.  The colon (:) was removed from Corresponding author | Line: 4,7,9,15  Page: 1 |

2. Reply to Reviewer 1’s comments:

| **Reviewer Number 1 comment number** | **Original comments of the reviewer** | **Reply by the author(s)** | **Changes done on page number and line number** |
| --- | --- | --- | --- |
| 1 | Combine the method subsections in the abstract (design, participants, and main outcome and measures) into a single subsection titled “Methods.” | Thank you for your comment, the change has been implemented | Line: 31-34  Page: 2 |
| 2 | contextualize the study by referencing existing literature on awareness of hearing loss in Jordan and globally. | Thank you. Further references were cited in the introduction as suggested. | Line: 95-107  Page: 5 |
| 3 | Highlight gaps in the current evidence base. | The gaps in relation to general population knowledge about HL and ear health have been highlighted | Line: 91-92, 106-107  Page: 4,5 |
| 4 | Specify inclusion and exclusion criteria. | Inclusion and exclusion criteria were added. | Line: 149-152  Page: 7 |
| 5 | It is mentioned that the study questionnaire was adopted from the literature. One of the referenced studies has English origin. Address how the study questionnaire, adapted from literature, was translated to Arabic and validated. | Thank you. This has been addressed in the newly created sub-section about the questionnaire. | Line: 190-195  Page: 9 |
| 6 | Create a dedicated subsection describing the questionnaire’s content, the number of questions, their nature, and the required participant responses. | Thank you. The sub-section has been included. | Line: 161-195  Page: 8,9 |
| 7 | Provide the exclusion rate | Thank you. This has been included in the methods section. | Line: 160  Page: 8 |
| 8 | Define how participants were categorized as having normal or impaired hearing. | This has been defined in table 3 in the results section and in the discussion section. | Line: 346-348  Page: 18 |
| 9 | Explain why most participants had normal hearing. Acknowledge that ensuring representation of individuals with hearing loss or family history of hearing impairments can be challenging due to their lower numbers compared to those with normal hearing and no family history of hearing loss. | Thank you. This has been addressed in the discussion section. | Line: 346-352  Page: 18 |
| 10 | Report both numbers and percentages in the results. | Done throughout the results section | Results section |
| 11 | Detail the training of personnel administering the instruments. | This information has been added in the methods section. | Line: 153-157  Page: 7-8 |
| 12 | Address missing data and how it was handled in the analyses. | This now has been addressed in the methods section. | Line: 157-160  Page: 8 |
| 13 | Add a title to the discussion section. | A title to the discussion section was added. | Line: 292  Page: 16 |
| 14 | Discuss the robustness of the results. | This now has been addressed in the discussion. | Line: 346-348  Page: 18 |
| 15 | Elaborate on educational levels and hearing loss incidences in Jordan and explain how the authors generalize their findings to the broader Jordanian population. | Thank you. More details about educational levels and generalizing the findings were added in the discussion. | Line: 310-318  Page: 16-17 |
| 16 | Explore the external validity of the study’s conclusions. | The external validity has been addressed in the conclusion section. | Line: 404-407  Page: 21 |
| 17 | Compare the study’s results with related research on hearing loss awareness in Jordan, such as Alqudah et al.'s work (Parental knowledge and attitudes towards hereditary hearing loss and genetic technology). | Thank you. That reference has been added and related research has been discussed. | Line: 314-316  Page: 16-17 |

3. **Reply to Reviewer 2’s comments:**

| **Reviewer Number 2 comment number** | **Original comments of the reviewer** | **Reply by the author(s)** | **Changes done on page number and line number** |
| --- | --- | --- | --- |
| 1 | Title: The title is clear and concise but could be more engaging. Consider rephrasing to "Exploring Awareness of Hearing Loss and Ear Health in Jordanian Adults", to enhance its appeal to a broader audience.  Abstract: The abstract effectively summarizes the study, but it could benefit from adding more specific numerical results to highlight key findings (e.g., the percentage of respondents aware of certain facts). Additionally, mention the most common misconceptions identified in the study. | Thank you for your suggestion regarding the title, it has been changed accordingly  The abstract has been updated as per the recommendations. | Line: 1, 39-46  Page: 1, 2 |
| 2 | Contextualization of Hearing Loss: The introduction should include more global data on the consequences of hearing loss, particularly its impact on quality of life, to provide broader context for the study.  Emphasize Preventive Strategies: While the introduction discusses the consequences of hearing loss, it could benefit from an expanded section on preventive strategies and how awareness can lead to early intervention. | Thank you. The recommended additions have been implemented in the introduction section. | Line: 95-115  Page: 5 |
| 3 | Sampling Details: The systematic sampling method could be explained more thoroughly. For example, describe why every third person was chosen and whether this approach ensured a representative sample of the Jordanian population.  Statistical Justification: Expand the section on statistical analysis to provide more detail about how the choice of tests (e.g., t-tests, ANOVA) was justified given the study design. | Thank you. The explanation about the sampling method has been added, as well as the justification for using the statistical tests. | Line: 145-149, 202-209  Page: 7, 10 |
| 4 | Interpretation of Findings: Although the results are well-presented, the discussion around gender differences in awareness could be expanded. Specifically, explain why women demonstrated higher levels of awareness in particular domains, linking it to cultural or societal roles in Jordan.  Detailed Misconceptions: Highlight the most concerning misconceptions more prominently in the results. For example, the incorrect belief about vocal tract defects causing deafness/muteness should be discussed in greater detail. | Thank you for highlighting this point. The discussion has been updated, specifically about women.  More details about misconceptions have been discussed. | Line: 319-330, 331-345  Page: 17-18 |
| 5 | Comparison with Other Studies: The discussion should draw more explicit comparisons between the results of this study and findings from similar studies in other Middle Eastern countries. This would help contextualize the findings and demonstrate the broader applicability of the results.  Link to Broader Health Education: Discuss how these findings could influence broader public health campaigns in Jordan. How might these results inform policy changes or educational interventions in schools or workplaces? | The recommended comparisons, the broader applicability and link to broader health education have now been addressed. | Line: 314-318, 325-330, 338-345  Page: 16, 17, 18 |
| 6 | Actionable Recommendations: The conclusion could benefit from more specific recommendations for future public health campaigns, such as targeting certain misconceptions or focusing on at-risk populations (e.g., men, less educated individuals).  Future Research: Suggest areas for further research, particularly in rural populations or among different age groups, to identify whether similar trends in awareness are present across all demographics in Jordan. | Thank you. The recommendations were included in the conclusion section and within the discussion section. | Line: 316-318, 328-330, 343-345, 363-367, 387-395  Page: 17, 18, 19, 20 |
| 7 | Recent Studies: Include more recent references that focus on public awareness of hearing loss and ear health, particularly in developing countries. This will make the study’s context more relevant and up-to-date.  you may consider use the following recommendation to support your discussion : Nashwan, Abdulqadir J., Valdez, Glenn Ford D., Villar, Ralph C., et al. Stigma towards health care providers taking care of COVID-19 patients: A multi-country study. Published: April 2022 in Heliyon DOI: 10.1016/J.HELIYON.2022.E09300  This article explores health-related stigma, which can be used to highlight how stigma or misconceptions about hearing loss and ear health may affect public awareness and healthcare-seeking behavior. Shaban, Marwa Mamdouh, Sharaa, Heba Magdy, Shaban, Mostafa, et al. Effect of digital-based nursing intervention on knowledge of self-care behaviors and self-efficacy of adult clients with diabetes. Published: February 2024 in BMC Nursing DOI: 10.1186/S12912-024-01787-2  This study provides insights into how educational interventions improve health awareness, supporting the idea of public education campaigns for hearing loss and ear health. Shaban, Mostafa, Mohammed, Huda Hamdy, Ibrahim, Ateya Megahed, et al. Exploring the nurse-patient relationship in caring for the health priorities of older adults: qualitative study. Published: July 2024 in BMC Nursing DOI: 10.1186/S12912-024-02099-1  This article discusses the role of healthcare providers in raising patient awareness, which can relate to the role of audiologists and healthcare professionals in enhancing ear health awareness. Bakhsh, Ebtisam, Shaban, Mostafa, AlSheef, Mohammed, et al. Exploring the clinical efficacy of venous thromboembolism management in Saudi Arabian hospitals: An insight into patient outcomes. Published: April 2023 in Journal of Personalized Medicine DOI: 10.3390/JPM13040612  This article emphasizes the importance of effective management and patient awareness, which parallels the necessity for increased awareness and proper management of hearing loss in Jordan. | Thank you for your valuable suggestions. All the suggested references were cited in the discussion section and have been added as references: 29, 35, 39,42 | Line: 316-318, 362-363, 389-390, 393-395,  Page: 17, 19, 20 |
